# Comparing Surgical Site Infection and Scar Cosmesis Between Conventional Linear Skin Closure Versus Purse-string Skin Closure in Stoma Reversal - A Randomized Controlled Trial

**DOI:** 10.7759/cureus.2181

**Published:** 2018-02-11

**Authors:** Sathasivam Sureshkumar, Kunnathoor Jubel, Manwar S Ali, Chellappa Vijayakumar, Anandhi Amaranathan, Sudharsanan Sundaramoorthy, Chinnakali Palanivel

**Affiliations:** 1 Surgery, Jawaharlal Institute of Postgraduate Medical Education and Research (JIPMER), Puducherry, India.; 2 Surgery, AIIMS, Bhubaneswar, India; 3 Preventive Medicine, Jawaharlal Institute of Postgraduate Medical Education and Research (JIPMER), Puducherry, India.

**Keywords:** stoma reversal, purse string, linear skin closure, scar cosmesis, patient satisfaction, quality of life, stoma closure, conventional technique

## Abstract

Introduction

Stoma closure is one of the most frequently performed surgeries. The common complications are surgical site infection (SSI) and poor scar cosmesis. Purse-string sutures are expected to have less incidence of SSI due to the free drainage of secretions from the wound and possibly the early detection of a wound infection. Compared to the conventional linear closure, the purse-string closure technique is expected to have less wound infection, improved scar cosmesis, and good patient satisfaction because of a smaller size scar. Hence, a well-structured study is required to substantiate the advantage of this technique.

Methodology

This randomized control trial was carried out for two years in a tertiary care centre in Southern India. Patients with various stoma reversals, including colostomy, as well as ileostomy reversal, were included in the study. Patients were divided into Group I - conventional linear skin suturing (n = 40) and Group II - purse-string closure (n = 40). After the closure of rectus muscle, the skin is closed using the purse-string method (subcuticular) in the experimental group.

Results

Both the groups were comparable with respect to age, gender, body mass index (BMI), the presence of co-morbidities, and indication for surgery. Stomal procedures were done (26.3%) for malignant cases. The difference in mean hospital days for both groups were statistically insignificant (11.95 vs. 9.9; p = 0.927). The incidence of SSI between the groups were statistically significant (17 vs. 3; p = 0.003). The mean Patient and Observer Scar Assessment Scoring (POSAS) scores between the groups (65.30 vs. 83.40; p = 0.012) were statistically significant. This proved significant improvement in scar cosmesis in purse-string skin closure. At one month postoperative, the purse-string group had better patient satisfaction (3.08 vs. 4.48; p = 0.001), which was evidenced by a mean Likert 3 scale score. The mean visual analogue scale (VAS) score did not show any significant difference in pain between the groups.

Conclusion

Purse-string skin closure for stoma reversal had significantly less incidence of SSI. The duration of antibiotic therapy was also less in purse-string skin closure patients as compared to linear skin closure patients. Purse-string skin closures significantly improved the scar outcome and patient satisfaction.

## Introduction

Stoma reversal surgeries are complicated by many factors, including macerated skin, surgical site infection (SSI), and outsized scar, which leads to less patient satisfaction [1]. Many of these factors influencing the outcome are addressed by including antibiotic prophylaxis in the pre- and postoperative period, using monofilament suture material, etc. However, the wound infection rate remains high with a poor cosmetic outcome. Skin closure technique like purse- string sutures may be useful in addressing these issues.

Purse-string sutures are expected to have less incidence of SSI due to the free drainage of secretions from the wound and possibly early detection of wound infection. Compared to the conventional linear closure, purse-string closure is expected to have a smaller size scar. However, other factors, such as operating time, blood loss, and postoperative pain, are also to be considered [2-5]. Hence, a well-structured study is required to substantiate the advantage of this technique.

The few studies that have tried to assess the advantage of one technique over the other found that wound infection is less in purse-string closure. However, the other aspects, including scar cosmesis and patient satisfaction, are not well evaluated [3-6]. So far, no such study has been conducted from India to analyse the effectiveness of the purse-string skin closure. Hence, this study was conducted to assess the effect of purse-string closure in reducing the wound infection and to improve cosmesis.  

## Materials and methods

Study design

This study was conducted as an open-labeled, parallel arm, prospective randomized control trial in a tertiary care centre in South India over a period of two years. Informed consent was taken from all patients who participated in the study after explaining the nature of the study, methodology, and risks involved in the study. Information regarding all participants of the study was kept confidential. Patients were given freedom to withdraw from the study at any point in time. The study was conducted in lines of the declaration of Helsinki. The objectives were to compare the rate of SSI, scar cosmesis, Quality of Life (QOL), postoperative pain, and patient satisfaction between the two procedures.

Study participants

The study included all patients undergoing stoma closure in the Department of Surgery irrespective of the indication for the index (first) surgery. Patients less than 12 years of age (pediatric patients) and patients not willing for participation were excluded from this study. After consent, patients were randomized to Group I (conventional linear skin closure) or Group II (purse-string skin closure).

Sample size calculation

Assuming 95% confidence level (alpha = 0.05), 80% power, 40% as the proportion of patients developing wound infection after the linear closure method [1-3], and the expected proportion of wound infection after the purse-string method as 10%, the sample size required was calculated as 40 in each group (Open Epi, Version 3).

Randomization

Randomization was done using computer generated random numbers (using the block randomization technique) by a third person not involved in the study. Allocation concealment was ensured by the use of a sequentially numbered opaque sealed envelope.

Study procedure

**Figure 1:**
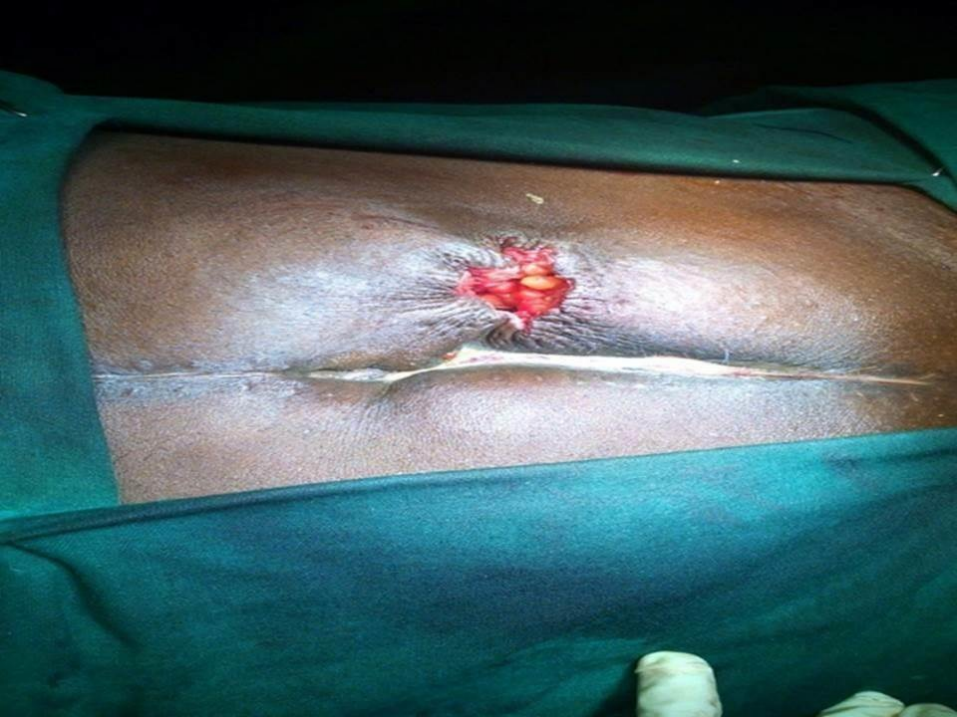
Purse-string closure method - immediate postoperative wound

All study patients received prophylactic antibiotics before the procedure unless contraindicated due to hypersensitivity, renal failure, etc. The incision for stoma takedown was made as an elliptical incision in Group I (linear skin closure) and as a circumstomal incision in Group II (purse-string skin closure). After adhesiolysis of the stoma, a simple closure or resection and hand-sewn end-to-end anastomosis or resection and stapled side-to-side anastomosis was performed at the discretion of the operating surgeon. A layer by layer linear suturing was performed on the fascia of the rectus abdominis muscle. Subcutaneous tissue was not sutured. At the time of wound closure in Group I, the elliptical incision was closed by a conventional linear skin closure method using vertical mattress interrupted sutures with a non-absorbable suture. In Group II, a circumstomal incision was approximated using a purse-string subcuticular (on the dermal layer) suture using an absorbable suture (Figure 1).

Parameters assessed

Baseline demographic parameters, such as age, gender, body mass index (BMI), and comorbidities, were assessed. The incidence of SSI was assessed based on the Centers for Disease Control (CDC) criteria and graded accordingly.

POSAS (Patient and Observer Scar Assessment Scale) was used to assess the wound scar cosmesis between the two groups in the present study. POSAS scale is a validated scale for comparing scar cosmesis which is comprised of two scales, one involving feedback from the clinician (observer scale) and the other taking into consideration the patient's feedback (patient scale). The observer scale takes into account six parameters, including vascularity, pigmentation, thickness, relief, pliability, and surface area, each of which are given scores from 1 to 10. The value 1 denotes normal skin and 10 for the worst possible scar, and the total score may lie anywhere from 6 to 60. The patient scale also takes into consideration six parameters, including pain, itching, colour change, stiffness, thickness, and irregularity of scar. Each of these is given scores from 1 to 10. The patient gives a score ranging from 1 indicating normal skin and 10 for the worst possible scar, and the total score ranges from 6 to 60.

**Figure 2:**
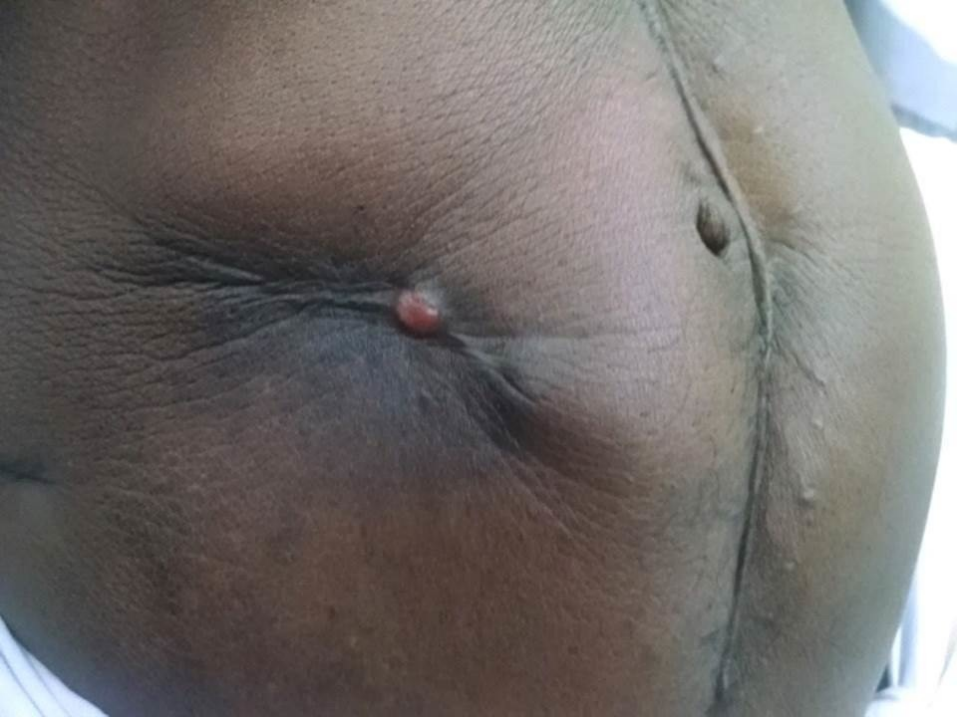
Purse-string skin closure scar three months post-stoma closure surgery

In our study, the sum of the observer scores and patient scores were taken as the ‘total score’ of the POSAS and were compared between the two groups to assess the difference in the scar outcome. Patients were assessed for scar cosmesis at four and 12 week follow-up periods (Figure 2).

Total operating time was calculated in minutes for comparison. Postoperative wound site pain was assessed in the immediate postoperative period and at 24 hours using the Visual Analogue Scale (VAS) score. QOL was assessed at the three month follow-up period by using the RAND 36-Item Short Form Health Survey, Short Form 36 version 2 (SF-36v2). Overall patient satisfaction was assessed using the Likert five-point scale, with five indicating ”excellent”, four “very good”, three “good”, two “fair”, and one “poor". 

Follow-up

Following discharge, patients were reviewed after one week for wound examination. In the linear closure group, sutures were removed on postoperative day 10. Patients were informed about warning signs of SSI, in which case they were to follow-up with the surgeon on an urgent basis. All patients were followed up on a regular basis up to three months following the procedure to assess the outcome parameters. 

Statistical analysis

The percentage of wound infections and proportions were analyzed using the Chi-square test. Other independent variables, such as age, sex, and comorbidities, were also assessed using Chi-square test. Continuous variables, such as operating time, POSAS scoring, and QOL scoring were assessed using the unpaired t-test when it follows a normal distribution. In the case of non-continuous variables, as well as continuous variables, not following a normal distribution, appropriate statistical tests were used.

## Results

A total of 80 patients were enrolled in the study, 40 each in the linear closure group and purse-string closure group. There was no mortality among the patients included in the study. The distribution of comorbidities, BMI (23.88 vs. 23.72; p = 0.0734), and age (50.1 vs. 43.4; p = 0.070) were comparable in both groups. The American Society of Anesthesiologists (ASA) fitness was also comparable in both groups (Table 1).

**Table 1:** Baseline Demographic Parameters Between Linear Closure Group and Purse-string Closure Group n: number; BMI: body mass index; ASA: American Society of Anesthesiologists

Demographic parameters		Linear closure (n = 40)	Purse-string closure (n = 40)	p-value
Mean age (Years)		50.1	43.4	0.070
Sex (n (%))	Male	28 (70%)	20 (50%)	0.0679
	Female	12 (30%)	20 (50%)	
BMI (Mean)		23.88	23.72	0.0734
Comorbidities (n)	Diabetes	6	5	-
	Hypertension	2	2	-
	Asthma	1	1	-
ASA I (n (%))		31 (77.5%)	36 (90%)	0.11
ASA II (n (%))		9 (22.5%)	4 (10%)	0.18

A stoma was created in elective surgery in 47 patients (59%), and the majority were operated upon for colorectal malignancy. Thirty-three patients (41%) underwent emergency stoma creation for various indications, including gangrene of the bowel, intestinal obstruction due to malignancy, abdominal tuberculosis, and traumatic bowel injury (Table 2).

**Table 2:** Causes for Stomal Surgery Between Linear Closure Group and Purse-string Closure Group n: number

Indication for stoma	Linear closure (n = 40)	Purse-string closure (n = 40)	p- value
Elective (n (%))	25 (62%)	22 (55%)	0.52
Emergency (n (%))	15 (48%)	18 (45%)	0.78
Mean duration for stoma reversal (days)	106.4	101.8	0.233
Type of stoma: Ileostomy (n (%))	17 (42%)	15 (37%)	0.64
Type of stoma: Colostomy (n (%))	23 (58%)	25 (63%)	0.65

The mean waiting period for stoma closure between the two groups did not differ significantly (106 vs. 101; p= 0.23). In purse-string patients, 15 were ileostomy reversal and 25 were colostomy reversal. In the linear skin closure group, 17 were ileostomy reversal and 23 were colostomy reversal; both groups did not significantly differ with respect to the type of stoma.The difference in the mean waiting period for stoma reversal following index surgery was comparable in both the groups (106.4 vs. 101.8; p = 0.233).

The duration of stoma reversal surgery was less in the purse-string closure group compared to the conventional linear closure group with the mean difference of 7 minutes. However, the difference was statistically insignificant (149.5 vs. 142.13; p = 0.064). The incidence of SSI was significantly lesser in the purse-string closure group compared to the conventional linear closure group. Only three patients developed SSI in the purse-string closure group compared 17 in the conventional linear closure group, which was statistically significant (17 vs. 3; p = 0.003). All SSIs were superficial grade I and were treated by conservative management. None of the patients in either group developed deep or organ space SSIs. Two patients in the conventional linear closure group developed an anastomotic leak compared to one patient in the purse-string closure group, which was statistically significant (2 vs. 1; p = 0.0002). The mean antibiotic use was also significantly less in the purse-string closure group compared to the conventional linear closure group (6.08 vs. 3.8; p = 0.0002). Comparison of pain relief using the VAS score didn’t show any significant difference between the groups. The number of patients requiring additional morphine dosing after 24 hours post-surgery were higher in the conventional linear closure group compared to purse-string closure group. However, the difference was statistically insignificant (6 vs. 8; p = 0.5562) (Table 3).

**Table 3:** Comparison of Operating Time and Immediate Postoperative Parameters Between Linear Closure Group and Purse-string Closure Group n: number; SSI: surgical site infection; VAS: visual analogue scale; SD: standard deviation

Peri-operative outcome	Linear closure (n=40)	Purse-string closure (n=40)	p-value
Mean duration of surgery (min)	149.5	142.13	0.064
SSI (n (%))	17 (42.5%)	3 (7.5%)	0.003
Anastamotic leak (n (%))	2 (5%)	1 (2.5%)	0.0002
Mean antibiotic usage (days)	6.08	3.8	0.0002
VAS score – Immediate postoperative (mean (SD))	5.05 (1.4)	5.03 (1.39)	0.970
VAS score– 24 hours following surgery (mean (SD))	3.05 (1.28)	2.98 (1.29)	0.959
Number of patients injectable morphine after 24 hours (n (%))	6 (15%)	8 (20%)	0.5562

The conventional linear closure group patients (Group 1) stayed longer in the hospital compared to purse-string closure group patients (Group 2) who were discharged two days earlier on average than Group 1. However, the difference in the mean hospital days for both groups was statistically insignificant (11.95 vs. 9.9; p = 0.927). An increased length of the scar was noted in Group 1, but it failed to show any statistical significance with the p-value of 0.812. Hypertrophic scar was present in two patients of Group 1 (5% of incidence), whereas no scar complication was noticed in Group 2. In Group 1, four patients (10%) developed an incisional hernia, whereas none in Group 2 developed an incisional hernia, which was statistically significant with the p-value of 0.0003 (Table 4).

**Table 4:** Comparison of Hospital Stay and Late Postoperative Complications Between Linear Closure Group and Purse-string Closure Group n: number

Postoperative outcome	Linear closure (n = 40)	Purse-string closure (n = 40)	p-value
Mean hospital stay (days)	11.95	9.9	0.927
Mean scar length (mm)	53.13	45.03	0.812
Incisional hernia (n (%))	4 (10%)	0	0.0003

Out of 20 diagnosed SSI patients, five (25%) were culture-negative. More than 60% SSIs were due to gram-negative organisms, E. coli being the most prevalent one. Mixed growth was present in one out of 20 (5%) swabs. Fungal growth was present in two out of 20 (10%) swabs in both groups (Table 5).

**Table 5:** Organism Causing Surgical Site Infections Between Linear Closure Group and Purse-string Closure Group n: number

Organism	Linear closure (n = 40)	Purse-string closure (n = 40)
Gram-positive (n(%))	0	0
Gram-negative (n (%))	10 (50%)	2 (10%)
Mixed (n (%))	1 (5%)	0
Fungus (n (%))	1 (5%)	1 (5%)
Anaerobic (n (%))	0	0
Sterile (n (%))	5 (25%)	0

The mean POSAS scar cosmesis score between the groups was statistically significant, suggesting improvement in scar cosmesis in Group 2 (65.30 vs. 83.40; p = 0.012). QOL was assessed by SF–36v2 score between the two groups and the differences in the score were found to be statistically insignificant (51.75 vs. 52.98; p = 0.388). The patient satisfaction assessed by the Likert scale showed no difference at discharge. However, the Likert score at one week (2.98 vs. 4; p = 0.021) and one month (3.08 vs. 4.48; p = 0.001) following discharge showed significantly improved patient satisfaction in Group 2 compared to Group 1 (Table 6).

**Table 6:** Comparison of Quality of Life and Patient Satisfaction Parameters Between Linear Closure Group and Purse-string Closure Group POSAS : Patient and Observer Scar Assessment Scoring; SF-36v2 : Short Form 36, version 2; n: number

Quality of Life Parameters	Linear closure (n = 40)	Purse-string closure (n = 40)	p-value
POSAS score (mean)	65.30	83.40	0.012
SF – 36v2 score (mean)	51.75	52.98	0.388
Patient satisfaction likert score in postoperative period (mean)			
Likert 1 (at discharge)	3	3	-
Likert 2 (one week)	2.98	4	0.021
Likert 3 (one month)	3.08	4.48	0.001

When comparing the SSIs between patients who underwent ileostomy closure and colostomy closure in the purse-string closure group, no significant differences were found between the two subgroups (4 vs. 2; p = 0.278). Other outcome parameters, including VAS score, POSAS scar cosmesis score, QOL score, and patient satisfaction score, also didn’t show any significant difference between ileostomy and colostomy reversal subgroups (Table 7).

**Table 7:** Comparison of Outcome Parameters Between Patients Who Underwent Ileostomy Closure and Colostomy Closure in the Purse-string Closure Group n: number; SSS: surgical site infection; SD: standard deviation; POSAS : Patient and Observer Scar Assessment Scoring; VAS: visual analogue scale; SF-36v2: Short Form 36, version 2

Parameter Assessed	Ileostomy (n = 32)	Colostomy (n = 48)	p-value
SSI (n (%))	4 (13.3%)	2 (4%)	0.278
VAS Score (mean (SD))	5.4 (1.55)	4.8 (1.26)	0.384
Likert Score (mean (SD))	2.8 (1.32)	3.12 (1.33)	0.968
POSAS Score (mean (SD))	82 (9.61)	84.24 (12.28)	0.325
SF–36v2 Score (mean (SD))	53.53 (5.08)	52.64 (4.92)	0.895

## Discussion

Having an enterostoma significantly affects the patient’s QOL. SSI following stoma reversal is a significant issue, as having a persistent wound with a discharge from the local site. It creates much discomfort to the patient and may significantly affect the QOL even after the stoma reversal. Efforts have been taken to reduce SSI following stoma closure by assessing various factors. Types of skin closure techniques, including the purse-string closure, have gained much interest recently as it may have a significant impact on the wound healing.  

The mean age of patients undergoing stoma reversal in this study was 46.75 years, which was comparable with the study by Lee et al. where the mean age was 51 years [4]. In their study, Lee et al. noted that in patients belonging to ASA III the rates of SSI were higher when compared (33.3 % vs 4.8 %; p = 0.02) to those belonging to ASA II and ASA I [4]. Both groups in this study were comparable in terms of ASA classification and none of the operated cases belonged to ASA III stage. Obesity can significantly impair wound healing. It can make the surgery difficult due to the increased amount of subcutaneous fat. The mean BMI of our study population was 23.8, which falls within the normal range. The mean BMI between the two groups were comparable. Kaiser et al., in their assessment of morbidity and mortality after the closure of ileostomy, found that being hypertensive increases the chance of wound infection [5]. The present study didn’t show any such correlation. 

In the study of Reid et al., the mean duration for stoma reversal following index surgery was 135 days [7]; in the study by Lee et al., it was 124 days [4]. Delayed reversion often makes surgery difficult due to the matured adhesions at the stoma site, in addition to complications like skin maceration [7]. In this study, the mean gap between index surgery and stoma reversal was 104.1 days and the duration was comparable between the two groups. Both the above-mentioned studies included a greater proportion of patients with abdominal malignancies compared to our study. The requirement of chemotherapy and radiotherapy in the post-excision period could be the reason for the delay when compared to the present study.

In the study of Dusch et al., the mean duration of surgery was significantly less in the purse-string closure group [6]. In our study, no significant difference was found in the duration of surgery. Duration of surgery is affected by many factors. The patients in the Dusch et al. study had a homogenous population majority being operated upon for malignancy, expected to have the same level of difficulty intraoperatively, and the difference could be due to the purse-string closure taking considerably lesser time than the linear closure [6]. In our study where the patients underwent surgery for various indications, we had different levels of difficulty and the difference occurred possibly by the purse-string closure having had little contribution when considering the total operating time. In their meta-analysis on stoma reversal studies, Sajid et al. inferred that there was no significant difference in the duration of surgery between two types of stoma reversal [8].

In their study, Reid et al. observed the rate of SSI following linear skin closure for stoma reversal to be 39% and 7% in the purse-string closure, which showed a significant reduction [7]. Wound healing will be delayed in macerated skin. In linear skin closure for stoma reversal, the skin is closed primarily. This leaves no gap for the effluents from the wound to be drained. Moreover, the SSI will be diagnosed late compared to the purse-string closure method owing to the non-visibility of fascial planes. In the purse-string skin closure for stoma reversal, a gap usually (1-2 cm) is left in the center of the wound, which enhances the drainage of secretions from the wound. In our study also, the wound infection rate was 25%, which was comparable with other studies, and the incidence of SSI was found to be significantly less in the purse-string group. Smaller sub-centimeter purse-string gaps may be associated with increased rates of SSI, due to inadequate drainage as experienced by Reid et al. [7].

In the study of Bell et al., 11 patients were identified to have SSI [9]. Of these, positive cultures were found only in six patients (54.5%). Three were positive for Staphylococcus epidermidis, two showed Streptococcus pyogenes, and one showed growth of E. coli. Ferguson et al., in their document on identifying criteria for wound infection, emphasized the importance of clinical diagnosis of SSI [10]. In our study, the culture-positive rate was 75%, possibly due to better laboratory service and careful diagnosis of SSI. The majority of the culture-positive strains were E. coli-derived from faecal matter.

In this study, there was a significant reduction in the number of antibiotic days among the purse-string closure group probably because of a reduction in the rate of SSI. In the studies of Reid et al. and Sajid et al., the difference in the mean length of hospital stay was not significant between the two groups [7-8]. In the present study, the mean hospital stay between the two groups did not show any significant difference. The less severe grade of SSI, which include superficial incisional infections, are routinely managed conservatively with antibiotics and regular dressing. The majority of patients with superficial incisional SSI recovered without any significant morbidity. However, the higher grades of SSI, which include deep incisional or organ space infection, lead to significant morbidity and prolonged hospital stay due to the requirement of frequent debridement and specialised dressings. In the present study, all SSI were superficial incisional infections and were treated conservatively. None of our study population had deep incisional or organ space infections. This could be the possible reason for the insignificant difference in the hospital stay between the two groups as the superficial incisional infection has less impact on the length of hospitalization compared to the deep incisional or organ space infection. In the study of Lahat et al., the incidences of postoperative complications, excluding SSI, were 13.3% in the linear closure group, whereas the incidence of complications, excluding SSI, in the purse-string arm was 5.6% [11]. In our study, the overall incidences of postoperative complications were 10%. All cases of an incisional hernia were in the linear closure group and had postoperative SSI. SSI weakens the abdominal wall, which explains well the incidence of an incisional hernia among linear skin closure patients who had SSI.

Stoma reversal is done with a limited incision and is a less morbid procedure as compared to laparotomy. Routinely, study patients received intravenous analgesia from the immediate postoperative period until postoperative day 2. This could have affected the VAS score, and hence the two groups didn’t have much difference among the pain experienced. In the study of McCartan et al., there was no difference in pain between the groups [12].

The POSAS scale was developed for assessment of post-burn scars and at present is the best scale available for assessment of scar cosmesis. In this study, the mean POSAS score for the linear closure group was 65.30 and the mean POSAS score for the purse-string closure group was 83.40, implying the purse-string scar was better than the linear scar (p = 0.012). In the purse-string closure, wounds heal by secondary intention and the scab formed will cover the gap of the purse-string. This leads to a significant reduction in the scar formation. Moreover, in linear closure, the long incision with multiple sutures leads to poor cosmesis as well as increase the width of the scar. In the study of Marquez et al., the scar cosmesis was assessed by a visual scale and was comparable between the two groups [13]. The lack of patient feedback about the scar cosmesis, as well as a less comprehensive assessment of scar on an inferior scale, must have influenced their report.

In the study of Williams et al., the patient satisfaction was assessed using a four points score [14]. They inferred that patients with purse-string skin closure were very satisfied (70% vs. 20%) in comparison with the linear closure group. In their study, Klink et al. also used the Likert scale for assessment of patient satisfaction [15]. The better patient satisfaction in the late postoperative period among purse-string closure patients is possibly due to better scar outcome in the same, as evidenced by the POSAS score significantly favouring the purse-string group. SF-36v2 has been proven to be an effective tool for comprehensive assessment of QOL by many studies. The SF-36v2 consists of a questionnaire in eight components, including physical functioning, bodily pain, mental health, vitality, physical role functioning, emotional role functioning, social role functioning, and general health perception. The SF-36v2 assesses the overall QOL, including both the physical as well as the mental well-being of the patients. In the present study, we observed similar QOL outcomes between the linear and purse-string closure groups with comparable SF-36v2 scores. To live with a stoma is challenging for each patient and often has significant social implications on the ostomate’s life. Hence, reversal of stoma significantly improves their QOL. Compared to this fact, scar outcome and SSI would have had less influence on the overall QOL in the present study population. 

Limitations

This study involved stoma reversal for heterogeneous index surgeries. Even though effective randomization had been done to tackle this issue, it was desirable to have a homogenous population.

## Conclusions

The purse-string skin closure for stoma reversal has significantly less incidence of SSI and reduces the duration of antibiotic therapy compared to linear skin closure patients. The purse-string skin closure significantly improves the scar outcome and patient satisfaction compared to the conventional linear skin closure for stoma reversal.
